# Dynamic MRI of plantar flexion: A comprehensive repeatability study of electrical stimulation-gated muscle contraction standardized on evoked force

**DOI:** 10.1371/journal.pone.0241832

**Published:** 2020-11-05

**Authors:** Xeni Deligianni, Anna Hirschmann, Nicolas Place, Oliver Bieri, Francesco Santini

**Affiliations:** 1 Division of Radiological Physics, Department of Radiology, University Hospital Basel, Basel, Switzerland; 2 Department of Biomedical Engineering, University of Basel, Allschwil, Switzerland; 3 Department of Radiology, University Hospital Basel, Basel, Switzerland; 4 Institute of Sport Sciences, University of Lausanne, Lausanne, Switzerland; University of Pittsburgh, UNITED STATES

## Abstract

Quantification of skeletal muscle contraction in Magnetic Resonance Imaging (MRI) is a non-invasive method for studying muscle motion and deformation. The aim of this study was to evaluate the repeatability of quantitative measures such as strain, based on single slice dynamic MRI synchronized with neuromuscular electrical stimulation (NMES) and standardized to a similar relative force level across various individuals. Unilateral electrical stimulation of the triceps surae muscles was applied in eight volunteers during single-slice, three-directional phase contrast MRI acquisition at a 3T MRI scanner. To assess repeatability, the same process was executed on two different days by standardizing the stimulation aiming at evoking a fixed percentage of their maximal voluntary force in the same position. Except from the force, the effect of using the current as reference was evaluated on day two as a secondary acquisition. Finally, the presence of fatigue induced by NMES was assessed (on day one) by examining the difference between consecutive measurements. Strain maps were derived from the acquired slice at every time point; distribution of strain in the muscle and peak strain over the muscle of interest were evaluated for repeatability. It was found that fatigue did not have an appreciable effect on the results. The stimulation settings based on evoked force produced more repeatable results with respect to using the current as the only reference, with an intraclass correlation coefficient between different days of 0.95 for the former versus 0.88 for the latter. In conclusion, for repeatable strain imaging it is advisable to record the force output of the evoked contraction and use that for the standardization of the NMES setup rather than the current.

## Introduction

Quantification of skeletal muscle contraction in magnetic resonance imaging (MRI) can give valuable insight into muscle motion. The contraction can be either voluntary or evoked through neuromuscular electrical stimulation (NMES), which are different not only in terms of physiological response of the muscle (e.g. motor unit recruitment), but also in terms of practical details of their use in muscle diagnostics, rehabilitation and training. While voluntary contraction follows the Henneman size principle (i.e., small diameter motor units are recruited at lower force levels as compared to larger motor units) [1, 2], standard NMES induces a non-selective and mostly superficial random motor unit recruitment, allowing a greater contribution of type II muscle fiber at low force levels [1, 3–6]. Given the peculiarities of motor unit recruitment under NMES [4], NMES is a useful tool both for patients and for athletes in rehabilitation. Firstly, if a patient is unable to produce force levels high enough for a dynamic MRI muscle examination, NMES use can be a viable alternative [7, 8]. Secondly, since it follows a different activation pathway than voluntary contraction, it can yield complimentary information to examinations during voluntary exercise even in healthy individuals [9].

Muscle exercise in MRI can be visualized with various methods. Phase contrast (PC) MRI [10–12], amongst other techniques such as spin-tagging [13–15] and DENSE imaging [16], allows the dynamic acquisition of velocity and displacement images of various tissues, including the skeletal muscle. As imaging is usually not fast enough to be performed in real time (although there are reports of usage of this method, at the cost of lower spatial and temporal resolution [17]), a prospective or retrospective gating is usually implemented, under the assumption that the motion being quantified is occurring in a repetitive cycle through the same arc of motion. In this case, a portion of the k-space is acquired for every cycle and then the acquired datasets are reordered in a single period. For this reason, PC imaging requires the acquisition of several flow-sensitized images for each temporal frame of a periodic contraction.

Although there have been many promising studies [10, 11, 18–22], the reproducibility of PC imaging over spin tagging has been questioned [23]. The error of phase contrast sequence was reduced with the proper settings such as the phase encoding direction [24, 25], but there has been no relevant study for motion guided by NMES.

PC velocity images can be used for the calculation of displacement maps and subsequently other biomechanical properties of the skeletal muscle, which describe the deformation. In this way, a three-dimensional quantification of muscle motion is obtained in a non-invasive way. Information about the deformation behavior of the muscle during contraction is valuable, because it gives input for the condition of the muscle such as e.g. existence of stiffness or atrophy [13]. Two parameters are mainly used for the final contractility evaluation: the strain [26] and the strain rate [13, 19]. The strain is a tensorial quantity, which is defined as change of length per unit length in each spatial direction of a material under stress with respect to its length at rest. The strain rate is the temporal derivative of the strain, and it is therefore an instantaneous measure that requires no reference state. It can either be calculated from the strain, or directly from the velocity field.

It has been shown that strain/ strain rate in leg muscles during voluntary or evoked isometric plantar flexion expresses age-related dependence [18, 27]. Lee et al. showed that unilateral lower limb suspension significantly influenced strain values [28]. Recently, a 3D PC scan protocol was presented for imaging of voluntary contraction [11]. Even though it has been shown that it is possible to visualize or yield meaningful quantitative contraction characteristics with MRI, the standardization of these measurements is needed before using these methods for diagnostic purposes.

The challenges of scanning standardization during voluntary contraction are the requirement for synchronization of the feedback loop with the MRI acquisition and the compliance of the subject with the exercise, which becomes even more important in case of patients [10–12, 19–21]. An alternative is to use NMES for external controlled triggering.

Recently, it has been shown that MRI of the thigh muscles during NMES is feasible and the resulting strain/strain rate significantly depend on the level of the applied current [29]. These results were shown on single slice acquisitions. Since it is well accepted that muscle motion is three dimensional, the general aim of the current study was to investigate if the results of a two-dimensional acquisition are sufficiently robust under rescan conditions and which NMES settings can improve reproducibility. Although the current used is a decisive factor, it might not be a sufficient reference to ensure similar repeatability conditions. Therefore, the induced force was used in this study as the main reference parameter [30] (in case of the isometric plantar flexion muscles, the force is expressed as resistance). The main goal of the present study was to investigate whether the force is a more robust reference parameter as the current for inducing a reliable periodic neuromuscular stimulation during MRI acquisition and finally if there is a muscle fatigue effect already during the acquisition. Finally, a preliminary control of how the results are affected by muscle size will be performed. This overall assessment is relevant for the translation of this method into clinical practice as it is crucial to quantify the inherent variability of the measurements induced by the acquisition method itself.

## Materials and methods

The Ethics Commission of Northwest and Central Switzerland (Ethikkommission Nordwest- und Zentralschweiz-EKNZ) approved the study and all participants gave informed consent. Eight healthy volunteers (5 male/ 3 female, mean age: 31.9 ± 8.1 years, mean weight: 80.7 ± 15.1 kg, mean height: 1.79 ± 0.11 m) who usually performed low to moderate physical activity according to the short international physical activity questionnaire (IPAQ) were included in this study. The volunteers were asked not to perform any vigorous activity the day of the examination. The scans were performed on one side only and exclusion criteria, additional to the standard MRI contraindications, were recent injuries or operations in the examined leg and systemic pathologies with an involvement of the musculoskeletal system (e.g. arthritis, neuromuscular/neurodegenerative diseases, etc.). Experiments were performed on two different days within a time period of maximum four months.

An MR(Magnetic Resonance)-compatible force sensor was used to record the NMES-induced force during the dynamic scan at the height of the forefoot with a sampling rate of 10 Hz [30]. The sensor was attached to an MR-compatible supportive construction, which was fixed to the scanner bed and in addition kept the foot in a fixed position, as this setup is meant for isometric motion. After placing the volunteer on the scanner bed, in the same supine position as during the scan and just before moving the bed inside the scanner bore, the maximum voluntary plantar flexion force (MVF) was measured by the sensor. The leg was examined in a neutral position with the knee and hip in full extension, while the ankle angle was 90°. Measurements were repeated two to four times with at least 20 s recovery time until the MVF variation was less than 10% between the last two trials.

A summary of the various dynamic acquisitions is given in Table 1.

**Table 1:** Summary of the experimental protocol and the various scans of day 1 and day 2.

Dataset ID	Main characteristic	DAY	SCAN	
D_0_	*Basic Dataset*	1^st^	1^st^	with aimed *force output* of approximately 15% of the MVF
D_for_	*Force Reference*	2^nd^	1^st^	with aimed *force output* equal to (DAY 1-1^st^ SCAN)
D_fat_	*Fatigue Check*	1^st^	2^nd^	acquired just after (DAY 1-1^st^ SCAN) to check for muscle *fatigue*
D_cur_	*Current reference*	2^nd^	2^nd^	with NMES current equal to (DAY 1-1^st^ SCAN). This was an optional scan.

**MVF**: Maximum Voluntary Force.

The MRI acquisition was performed on a clinical 3T MRI scanner (MAGNETOM Prisma, Siemens Healthcare, Erlangen, Germany). The synchronization of the dynamic image acquisition and the electrical stimulation was accomplished as in [29]. In short, the NMES device was set to “synchronous mode,” where its two channels deliver stimulation at the same time. While the device remained outside the scanner room, one channel was guided to the room and attached through two electrodes to the subject’s skin for stimulation, while the other was fed as input to an Arduino custom made circuit [29] and used to generate the trigger signal for the MRI data acquisition.

For the triceps surae muscle belly stimulations, two rectangular electrodes were positioned over the gastrocnemii (below the popliteal fossa) and soleus (above the calcaneus) muscles. In order to identify the position of the electrodes (5.1 x 8.9 cm^2^ rectangular self-adhesive gel-based NMES electrodes [TensUnits.com, USA]) on the muscle belly of the calf, the subjects were asked to perform plantar flexion while standing prior to entering the scanner room. For localization purposes, glycerin markers were placed on the surface of both electrodes and a parasagittal slice through the markers was acquired.

The plane of data acquisition was decided based on the position of the two markers and in tendentially sagittal orientation. An out-of-phase gradient echo single slice image (voxel size: 1.125 x 1.125 x 3 mm^3^) was acquired to be used as a reference for precise segmentation.

The two-dimensional (2D) three-directional phase contrast images (i.e., single slice with velocity sensitivities in the three spatial directions, in-plane and through-plane) were acquired with velocity encoding of 30 cm/s on all directions, TR = 8.1 ms, TE = 5.63 ms and resolution 1.6 x 1.6 x 5 mm^3^, bandwidth/ pixel = 765 Hz/ Px, flip angle = 7°, FOV = 225 x 300 mm^2^, matrix: 192 x 144, 1 k-space line per segment, parallel imaging factor 2 (Generalized Autocalibrating Partially Parallel Acquisitions (GRAPPA) [31], 24 integrated reference lines), acquisition time 2.1 min and 43 temporal phases. It should be noted that there is some additional dead time after release for triggering.

A standard digital NMES unit (EM49, Beurer GmbH, Germany) was used to induce periodic contractions. The NMES protocol consisted of 400 us, 80 Hz bipolar rectangular pulses. The stimulation current was raised until to the point that it induced a force equal to 15% of the participant’s maximum force, unless if the volunteer felt uncomfortable (i.e., in which case the stimulation current was reduced). Periodic stimulation pulses (750 ms contraction, 750 ms pause) were applied during the whole duration of dynamic image acquisition (appr. 2.1 min of repeated stimulation pulses for the acquisition of one slice). The stimulation current was kept constant during the whole acquisition.

For every PC dataset, the average force evolution over all contraction cycles was calculated. Subsequently, the percentage of difference with respect to the average force of the reference scan D_0_ was also calculated.

### Post-processing

**Fig 1:**
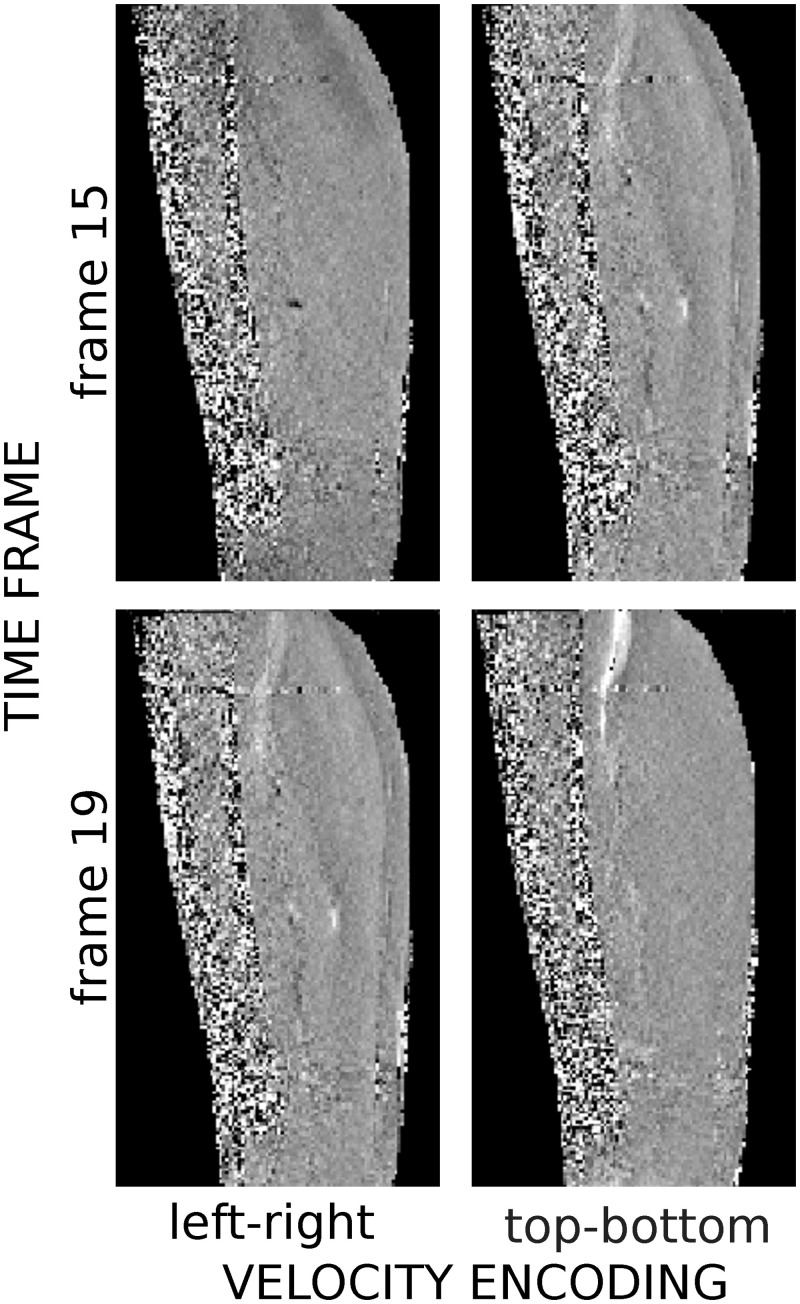
Example of raw velocity images with velocity sensitivity in the left-right (*left*) and top-bottom (*right*) directions (relative to the image) for two different frames at the first velocity peak (*top*) and at the time point where strain is maximum (*bottom*) are given.

For strain calculation, a region of interest (ROI) was drawn on an out-of-phase gradient echo image including both regions of the gastrocnemius and the soleus muscle. The velocity images were elaborated offline with Matlab (The Mathworks, Inc., Natick, MA, USA, R2018b). An example of velocity images at the first velocity peak (beginning of contraction) and at the maximum point for strain (approximately at the middle of the contraction) is given on Fig 1. Velocity images were corrected for phase shading, the displacement was calculated with forward/backward integration and the Langrangian strain was computed [29]. To increase precision the image grid was interpolated (factor of 9.9) spatially with a cubic interpolation using not-a-knot end conditions. The analysis was performed with the assumption that the pixels of the acquired slice do not move out of the slice during the acquisition. The tensors were diagonalized and the positive eigenvalue was considered as pixel-wise principal strain. The principal strain maps were calculated from the displacement maps and then visualized as described before [29], but the method of displacement derivative calculation was replaced with the addition of MaxPol for smoother numerical differentiation [32, 33]. In this case, a Selesnick differentiator was used [34, 35] and a two-dimensional steerable derivative kernel (15^th^ order tap polynomial, 3^rd^ degree polynomial controling the cutoff threshold at x- or y- axes, 1st order of differentiation).

**Fig 2:**
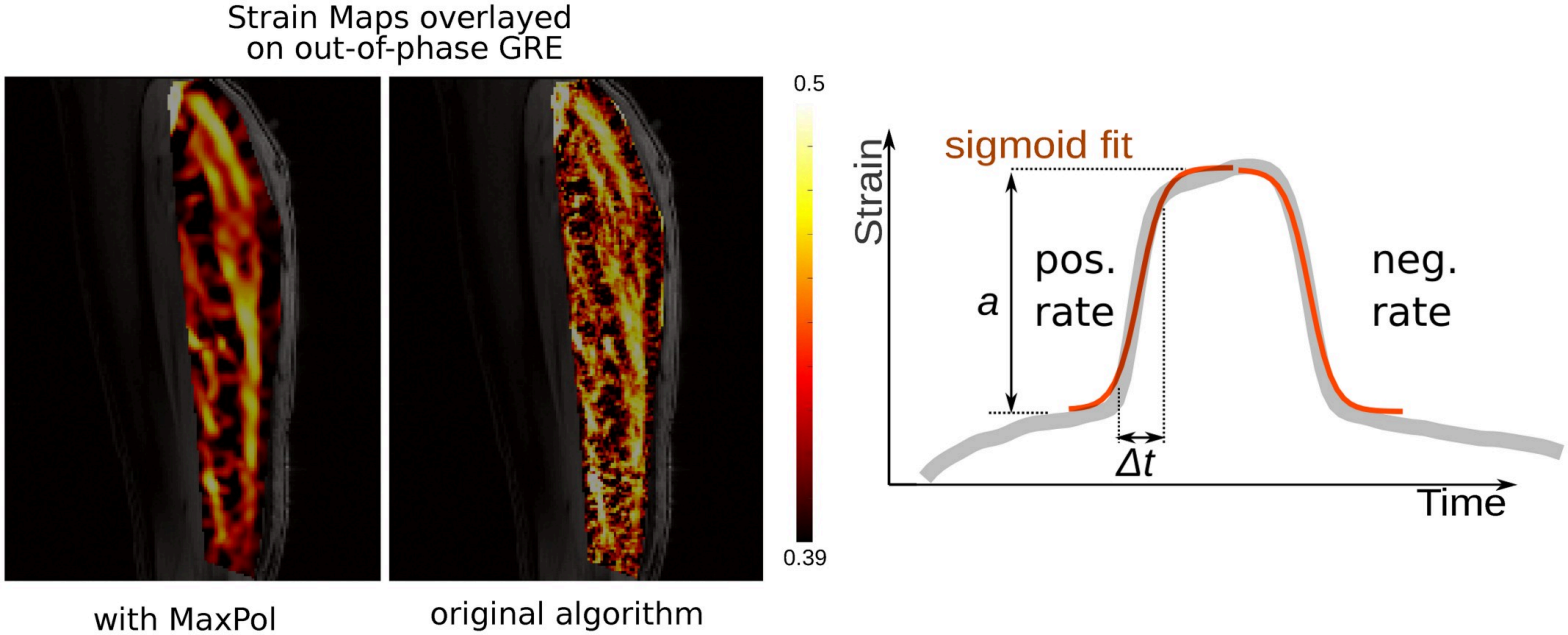
Example of a region-of-interest, a strain map with the MaxPlot algorithm and a strain map with the original postprocessing. On the strain graph, strain is given as a function of time, with overlayed explanation graphs explaining positive and negative rate of the strain.

As a representative value of strain, the spatial average over the ROI (see Fig 2) was calculated for every time frame of the reconstructed single contraction period and the maximum of this time curve was considered as peak strain. The rates at which the strain reached the maximum (*positive or buildup rate*) and relaxed to zero (*negative or release rate*) were also calculated by fitting a sigmoid curve to the corresponding portions of the strain curve (see Fig 2). The differences of the various rates were normalized to the maximum calculated rate amongst all data points.

### Statistical tests—Evaluation of repeatability & reliability

Similarity index (SI) was calculated in Matlab to compare strain maps on the middle of the contraction plateau (middle frame: 19^th^ frame). Affine registration was performed beforehand and then the calculation of the structural SI [36].

All statistical calculations and plots were produced using Matlab and R-studio [37]. The representative strain values of all eight volunteers were compared with scatter and boxplots. Three comparisons were performed following the scan protocol presented in Table 1; the basis scan D_0_ was compared to: a) D_for_, b) D_fat_, c) D_cur_.

To check for the repeatability by using the force as reference, D_0_ was compared to Df_or_ and respectively to check for the current as reference D_0_ was compared to D_cur_.

The two-way intraclass correlation coefficient (ICC) [38] was calculated to compare the first scans of each day D_0_ vs D_for_ and D_0_ to D_cur_ (*observations of exchangeable order*). The ICC was considered a measure of *reliability* since the measurements are exchangeable in order [39]. The repeatability coefficient (*RC*) was calculated as (39):

$$RC=1.96\cdot \sqrt{2\cdot }SD$$ [1]

where *SD* is the within-subject standard deviation. According to the respective theory, if the differences between two measurements are normally distributed (e.g., the differences of strain between successive scans) the absolute difference between two measurements on 95% of occasions is expected to not differ more than *RC*.

Finally, to check for potential muscle fatigue induced by NMES, D_0_ and D_fat_ were compared by using the concordance correlation coefficient since the measurements are non-exchangeable (cccrm package of R for Concordance Correlation Coefficient (CC) for Repeated Measurements by U-statistics was used).

## Results

To evoke a similar force, the current on day 2 was typically adapted by 1.5 mA (i.e., median difference), with the exception of two cases where the difference was 13 and 26 mA.

**Fig 3:**
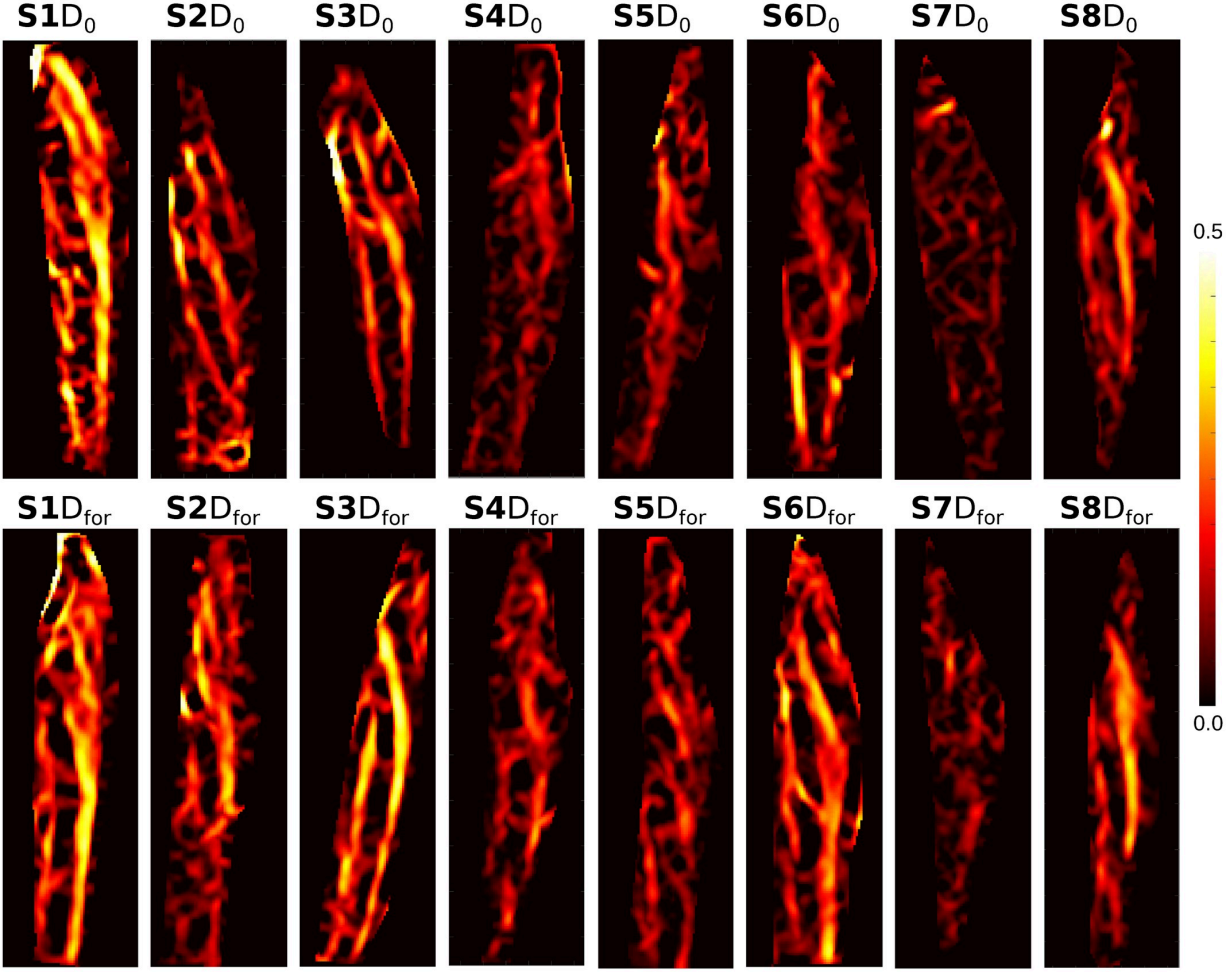
Strain-maps of a central frame from the first scan of day 1 (D_0_) from all the subjects from subject 1 (S1) to subject 8 (S8).

The strain maps of a time frame at the middle of contraction from the scan-rescan test (D_0_ vs D_for_) are presented in Fig 3. From a quantitative comparison, the average SI of all volunteers for scan-rescan strains with similar force output, denoted as SI(D_0_, D_for_), is in average 0.81 ± 0.02 comparable to 0.80 ± 0.03 of SI(D_0_,D_cur_) (i.e., identical current setup in different days). This is only slightly worse than 0.90 ± 0.03 of the SI(D_0_, D_fat_) (i.e., fatigue evaluation). With a few exceptions (e.g. subject 6 with SI(D_0_, D_for_) = 0.79 & SI(D_0_,D_cur_) = 0.78) the strain maps are qualitatively very similar (see Fig 3).

For the direct analysis of summarized strain values (i.e. maximum of the average value of the map, see Table 2) ICC/CC, RC and SD were compared. In order of increasing correlation and decreasing error, better repeatability and reliability is shown between consecutive measurements. A smaller ICC/CC and a larger error are found between scan-rescan measurements. The largest error and smallest ICC/CC are calculated between scan-rescan measurements setup based on current and not force. Finally, regarding the absolute strain values the SD was 0.01, which also gives the limit of precision.

**Table 2:** Reliability and repeatability coefficient of strain maps.

Strain	D_for_		D_fat_		D_cur_	
Median	0.092		0.083		0.067	
(Q1, Q3)	(0.069, 0.113)		(0.071, 0.091)		(0.053, 0.088)	
**D**_**0**_	***Scan-Rescan same force D***_***0***_ ***vs D***_***for***_		***Fatigue D***_***0***_ ***vs D***_***fat***_		***Setup with Current D***_***0***_ ***vs D***_***cur***_	
0.085	ICC	0.9524	CC	0.9957	ICC	0.8824
(0.068, 0.093)	CI	(0.784, 0.99)	CI	-	CI	(0.398,0.979)
	*p*-value	0.00015	*SE*	0.0026	*p*-value	0.00621
	RC	0.0288	RC	0.0059	RC	0.0372
	SD	0.0104	SD	0.0021	SD	0.0134
	$\bar{\left(D_{0}-D_{for}\right)}$	0.010	$\bar{\left(D_{0}-D_{fat}\right)}$	0.002	$\bar{\left(D_{0}-D_{cur}\right)}$	0.017

**Q1:** first quantile, **Q3:** third quantile, **ICC**: intra-class correlation coefficient, **RC**: repeatability coefficient, **CI**: Confidence Interval, $\bar{\left(D_{0}-D_{i}\right)}$: mean difference of D_0_ and D_i_

**Fig 4:**
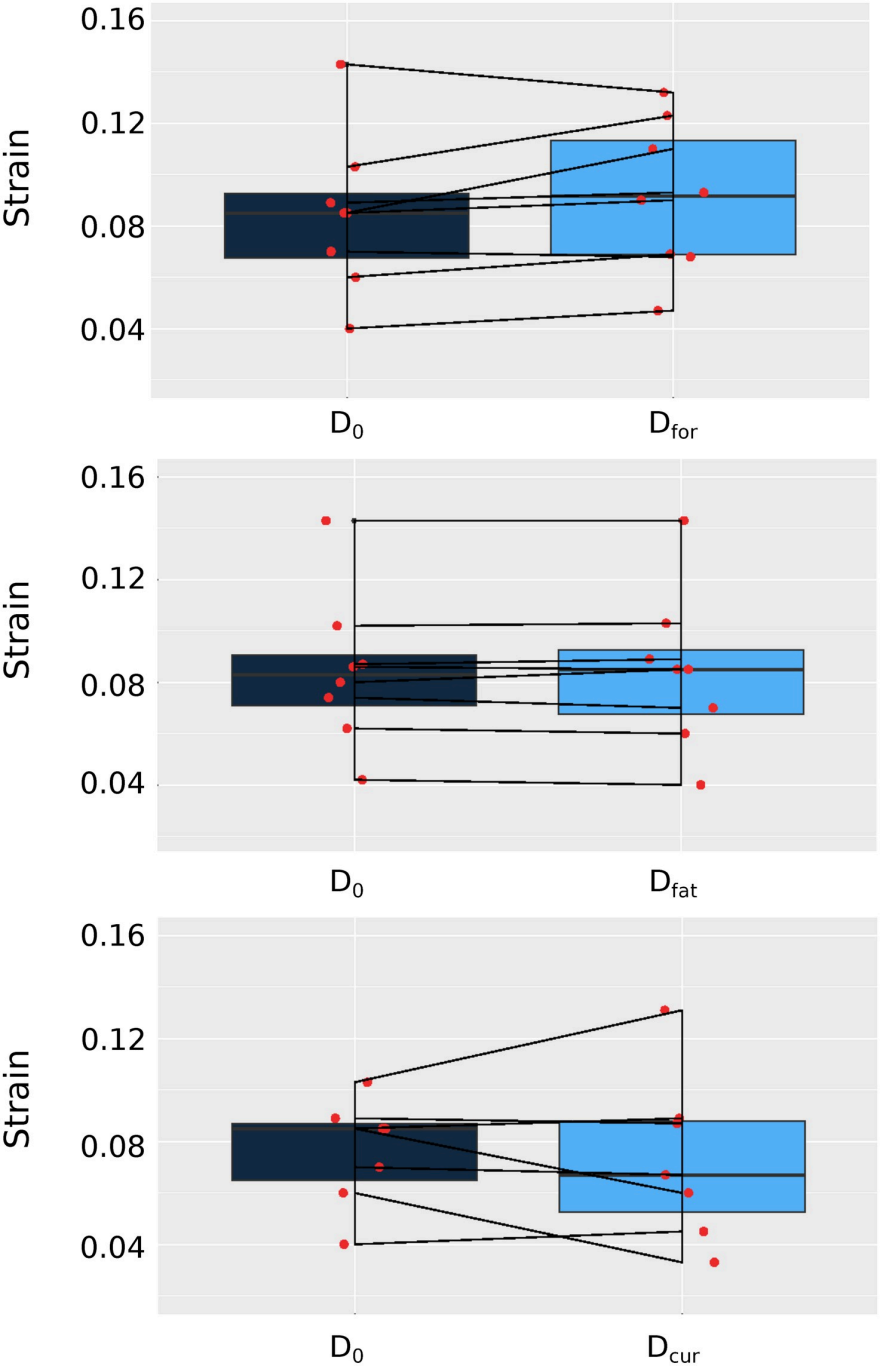
Boxplots of the maximum strain. (*top*) first scan of day 1 in comparison to first scan of day 2 which are setup at similar evoked force (D_0_-D_for_), (*middle*) the first scan of day 1 and a subsequent one to estimate fatigue (D_0_-D_fat_), (*bottom*) the first scans of day 1 and the second of day 2 which are setup at the same current (D_0_-D_cur_).

**Fig 5:**
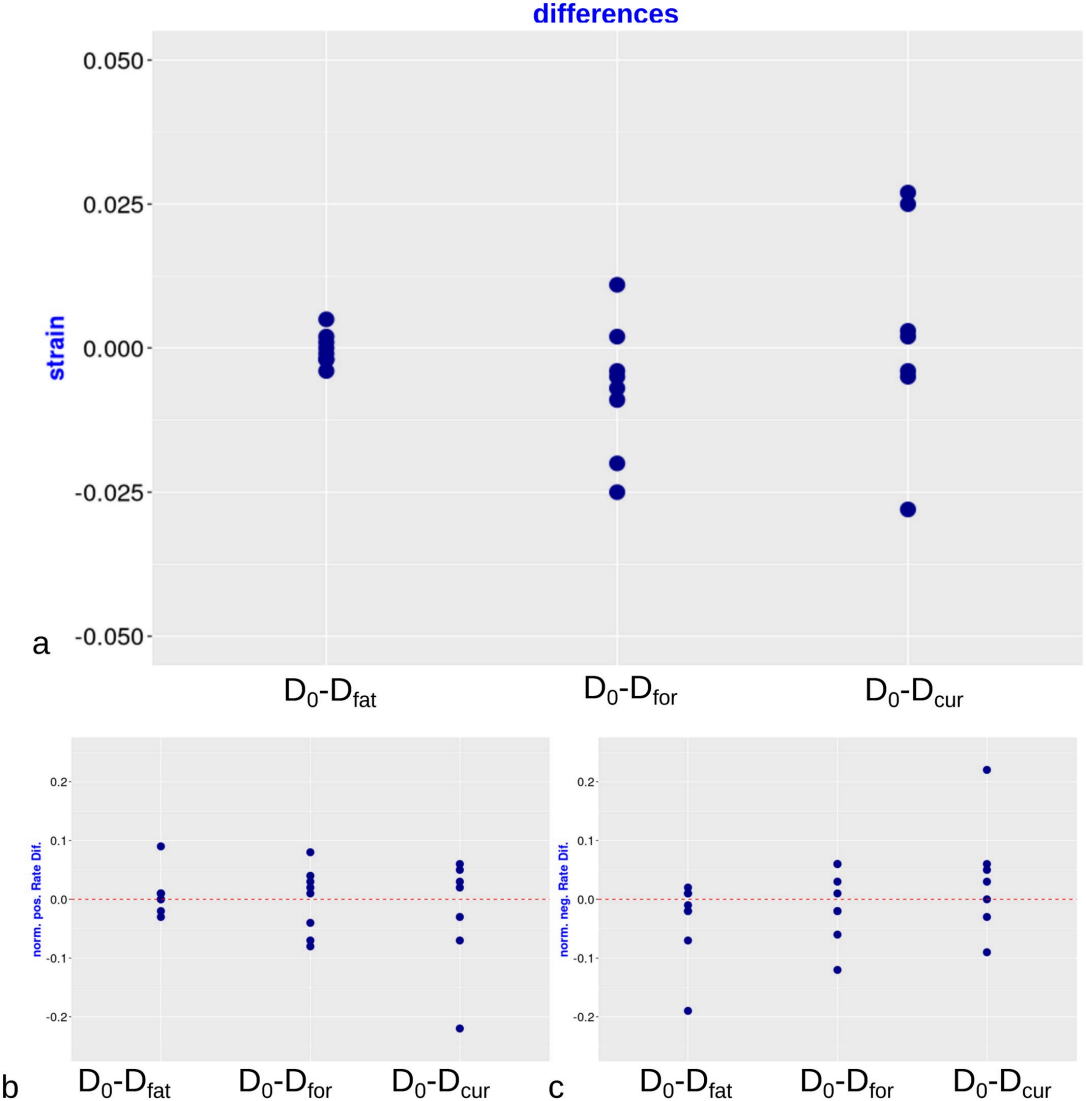
Scatter plots of differences. a. individual strain differences, b. normalized differences of the positive rate of the strain, c. normalized differences of negative rate of the strain between the repeated scan on day 1 (“fatigue effect”– D_0_-D_fat_), the scans on day 1 and day 2 with same force output (“same force”– D_0_-D_for_), and the scans on day 1 and day 2 with same stimulation current (“same current”– D_0_-D_cur_).

The individual maximum strains were compared with boxplots (Fig 4). As expected, overall the minimum difference was observed between consecutive acquisitions (i.e. no fatigue from the stimulation). Between rescans based on force or current, the differences are qualitatively similar. However, if instead of the absolute values, we visualize the differences for each individual (see Fig 5, *top*) there appears to be slightly less variability when the stimulation intensity is regulated according to exerted force in comparison to current (see Fig 5, *top*, central vs right column). In addition, the positive and negative rates of strain are less variable in the case of using the force as standardization factor (see Fig 5, *bottom*, central vs right column). However, the negative strain rate shows higher variability than the positive in the comparison of consecutive scans (see Fig 5, *bottom*, left column).

Ultimately, for every acquisition (*D*_*0*_, *D*_*fat*_, *D*_*for*_, *D*_*cur*_) the average of all rates across volunteers was calculated, which were lower for the positive rates (137.0 s^-1^, 130.5 s^-1^, 135.3 s^-1^, 133.6 s^-1^) than for the negative (163.1 s^-1^, 164.5 s^-1^, 169.3 s^-1^, 163.8 s^-1^). Overall, it was observed that there is little difference between the various sessions.

**Fig 6:**
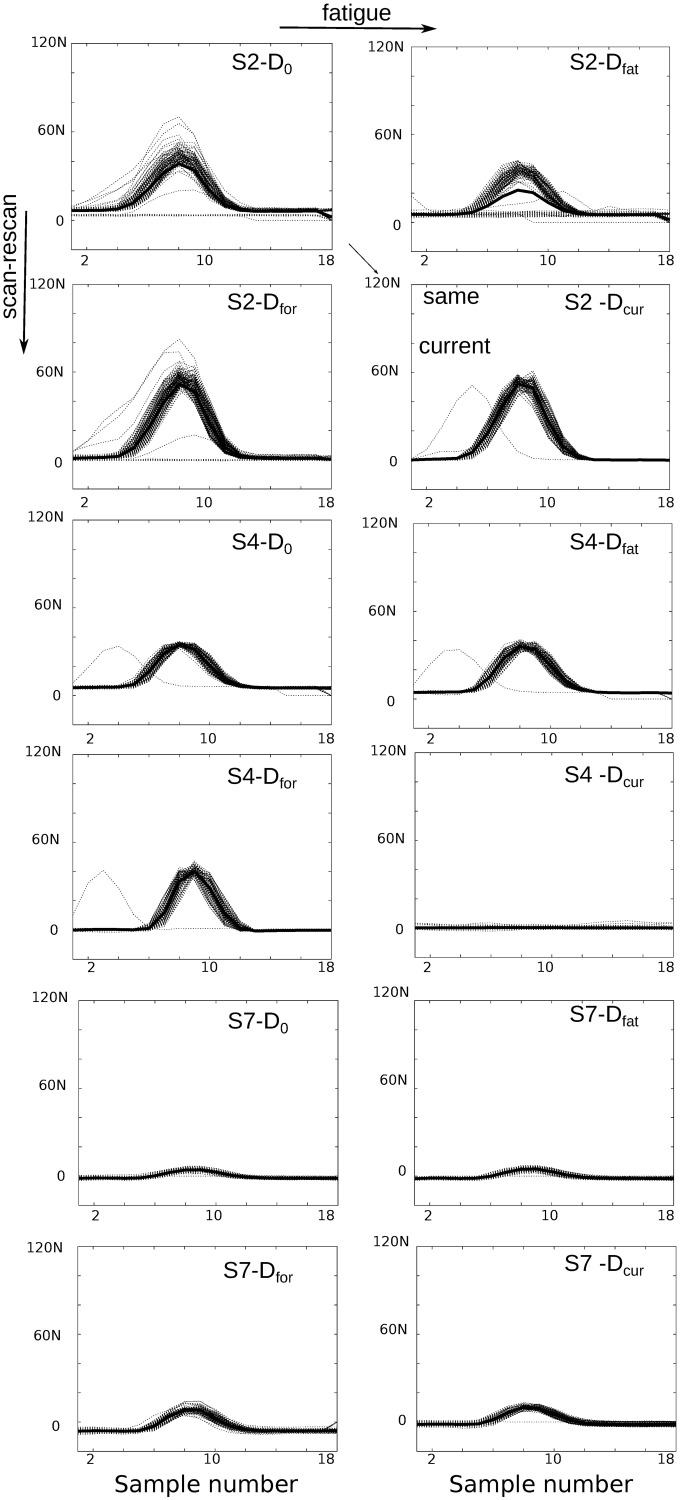
Evoked force during stimulation for three different cases. (*top*) higher resulting force, (*middle*) average, (*bottom*) low. In every window, the force curves for every stimulation are overlayed and on the top the average force during the scan.

Finally, examples of the curves of induced force are presented in Fig 6. The mean difference of the average scan force was maximum (43.8% (Q1/Q3 = 17.5/78.7%)) for D_0_-D_cur_, minimum for D_0_-D_fat_ (10.7% (Q1/Q3 = 8.7/32.5%)). For the scan-rescan tests D_0_-D_for_ the variation was in between (21.3% (Q1/Q3 = 14.3/44.3%)).

## Discussion

Established biomarkers for muscle characterization such as fat fraction and T_2_ relaxation are often indicators of an already progressed disease status and early detection of muscle alterations is very important [40]. The examined method of characterizing the condition of the muscle is simple, non-invasive, and of low additional cost on top of the cost of the MRI scan itself. However, since the settings of NMES are fundamental for the standardization of the evoked contraction [3, 7] and therefore of the MRI-based results, it is crucial to control the stimulation in the most repeatable way. The force output is employed for dynamic MRI of voluntary contraction [13], but to our knowledge there is no systematic study of a comparable setup for dynamic MRI during NMES.

In this study, we considered the effect of taking the evoked force or the applied current as a reference, as well as the influence of fatigue from continuously applying NMES. The results show that the evoked force during stimulation is the best reference for repeatable results. The effect of fatigue induced by this NMES protocol was minimal. It should be noted that in most cases only a minimal adaptation of the current was necessary to obtain the same force output, rendering the two control parameters de facto analogous. Nevertheless, in two cases the difference was large, which supports the fact the force is an important parameter to consider.

It should be noted that the sampling rate of the force is different (approximately two times longer) compared to the MR velocity acquisition. However, the two signals are fundamentally different, the force being recorded in real-time, whereas the MR velocity is acquired in a triggered fashion. The lower temporal resolution of the real-time force is in fact no limitation, as the actual dynamic of the force signal does not enter the analysis. It is not used for triggering the MR acquisition (in contrast to what was done in [41]), and it was in fact only recorded to ascertain the periodicity of the movement and the repeatability of the force output in the contracted state.

The remaining measurement variation of the quantitative strain measurements could be partly attributed to the three-dimensional nature of muscle motion. Undoubtedly muscle contraction is a three-dimensional effect and eventually a 3D analysis is important, but nevertheless this two-dimensional acquisition is certainly valuable since it is not time demanding. In the future, this limitation could be overcome by the application of advanced multi-planar or 3D imaging methods [11].

An alternative approach, not considered in this present study, could be the usage of spin tagging [42] methods instead of PC-MRI, which have shown good reliability for the detection of muscle motion [23]. However, Sinha et al [13] demonstrated good accordance between these two methods, and the fading of the tagging following T1 relaxation might render the choice of NMES stimulation protocols less flexible.

This study has some additional limitations. A factor that adds variability is the repositioning of the volunteer and of the electrodes. To properly evaluate repeatability, we would have had to repeat the scans, with the electrodes at the same position and with the use of longer breaks (i.e., to exclude fatigue). However, this was not possible due to time restrictions and we considered the implemented setup to be more representative of potential realistic conditions.

In this study including only young healthy volunteers the data showed quite homogeneous and repeatable results. In future investigations, various populations (e.g. in terms of age, sex and training) will be scanned as well, which may show a more stratified distribution of the assessed parameters. For example in a similar study [27], the response of senior volunteers was shown to be lower than in the younger cohort. In addition, strain needs to be evaluated in degenerative muscles to establish whether the proposed method can indeed differentiate between healthy and diseased muscle. Finally, dynamic muscle data from different age groups of healthy volunteers with normal daily activity is an essential first step in order to proceed on studying abnormal or pathophysiological muscle conditions.

In conclusion, this study shows that strain values produced with MRI of NMES-controlled evoked muscle contraction are highly repeatable when the induced electrically-evoked force during the scan is used as a reference. In this case the strain maps were not only qualitatively similar, but also the maximum strain values and the rates of strain increase, and release were very repeatable.

## Supporting information

S1 File (ZIP)

S1 Data (CSV)
